# Primary Ureteroscopy without Pre-Stenting for Proximal Ureteral Stones—Is It Feasible?

**DOI:** 10.3390/life13102019

**Published:** 2023-10-05

**Authors:** Alon Lazarovich, Rennen Haramaty, Asaf Shvero, Dorit E. Zilberman, Zohar A. Dotan, Harry Winkler, Nir Kleimann

**Affiliations:** 1 Department of Urology, Chaim Sheba Medical Center, Ramat Gan 5266202, Israel; rennenh@gmail.com (R.H.); asaf.shvero@sheba.health.gov.il (A.S.); dorit.zilberman@sheba.health.gov.il (D.E.Z.); zohar.dotan@sheba.health.gov.il (Z.A.D.); harry.winkler@sheba.health.gov.il (H.W.); nir.kleinmann@gmail.com (N.K.); 2 The School of Medicine, Faculty of Medicine, Tel Aviv University, Tel Aviv 6997801, Israel

**Keywords:** primary ureteroscopy, proximal ureteral stone, stone-free rate, ureteral stent

## Abstract

Background: Primary ureteroscopy with laser lithotripsy is the treatment of choice for distal ureteral stones. However, in cases of proximal ureteral stones, some urologists recommend the preliminary insertion of a ureteral stent and deferred ureteroscopy. We aimed to evaluate the necessity of preliminary ureteral stent insertion in the management of proximal ureteral stones by comparing the surgical outcomes of patients undergoing primary ureteroscopy with laser lithotripsy for proximal vs. distal ureteral stones. Methods: Medical records of patients who underwent ureteroscopy between 2016 and 2017 in our institution were retrospectively reviewed. Data collected included demographic data, stone size, renal function, intra- and post-operative complications, and stone-free rate (SFR). Patients were divided into two groups: proximal ureteral stones and distal ureteral stones. Results: The cohort included 241 patients who underwent ureteroscopy. Among them, 106 had a proximal ureteral stone. The median age was 51 (IQR 41–65) years. Patients who underwent ureteroscopy for proximal ureteral stones were significantly older (*p* = 0.007). The median stone’s maximal diameter was 7 (5–10) mm. The complication rate and stone-free rate (SFR) were similar in both groups (*p* = 0.657 and *p* = 1, respectively). The prevalence of post-procedural ureteral stent insertion was higher among patients who underwent ureteroscopy for proximal ureteral stones: 92.5% vs. 79.3% (*p* = 0.004). Conclusions: Our study concludes that primary ureteroscopy with laser lithotripsy for proximal ureteral stones is a valid and feasible treatment with a similar surgical outcome compared to distal ureteral stones. Preliminary ureteral stent insertion seems to be unnecessary.

## 1. Introduction

Ureteroscopy (URS) coupled with laser lithotripsy, conducted using either a flexible or semirigid ureteroscope, stands as the foremost approach for treating ureteral stones. As per the guidelines provided by the European Association of Urology (EAU) and the American Urological Association (AUA) regarding urolithiasis, the routine placement of a stent before ureteroscopy is not obligatory [1,2]. However, retrospective studies have demonstrated that pre-stenting can offer several advantages in the management of ureteroscopic procedures. These benefits include enhanced facilitation of stone management, cost reduction, reduced URS duration, improved stone-free rates (SFRs), and a decreased incidence of complications, particularly in cases involving larger or proximal stones [3,4].

Nevertheless, it is essential to recognize that pre-stenting necessitates an additional invasive procedure, typically performed under general anesthesia. Furthermore, it involves the presence of a stent within the patient’s urinary tract until the definitive stone treatment is completed. This may result in significant patient morbidity, including frequent urination, urgency, dysuria, pain, and hematuria [5]. In the case of small distal ureteral stones, a common surgical approach involves ureteroscopy without pre-stenting [6]. However, the question of whether to pre-stent for proximal ureteral stones remains a topic of ongoing controversy.

This study aims to assess the necessity of preliminary ureteral stent insertion for the management of proximal ureteral stones and to compare the surgical outcomes of primary ureteroscopy with laser lithotripsy for both proximal and distal ureteral stones.

## 2. Materials and Methods

Following approval from the institutional review board, we conducted a retrospective analysis of the medical records of patients who underwent primary ureteroscopy with laser lithotripsy for ureteral stones at our institution between 2016 and 2017. Primary ureteroscopy was defined as the performance of ureteroscopy with laser lithotripsy without prior ureteral stent insertion. 

**Inclusion and exclusion criteria:** We included patients 18 years old and above with ureteral stones. Patients with kidney stones and patients with ureteral stones who were pre-stented prior to ureteroscopy were excluded. 

**Data collection** encompassed various parameters, including demographic information, characteristics of the stone disease (side and location of the stone, maximal diameter, number of stones), pre-operative imaging modality, pre-operative serum creatinine levels, duration of symptoms prior to surgery, intra-operative details (bailout rate, post-operative drain—ureteral stent/open-ended catheter, use of a ureteral access sheath), office follow-up (stone-free rate, presence of residual stones), and any recorded complications.

**Ureteral stone’s location:** All patients underwent a pre-operative imaging test, either a non-contrast CT scan or renal ultrasound, in accordance with our institution’s standard practice. Patients in our cohort were categorized into two groups based on the location of their ureteral stones: distal and proximal. The distal segment of the ureter was defined as the portion extending from the lower part of the sacroiliac joint to the ureteral orifice. The proximal ureter was defined as the segment extending from the ureteropelvic junction to the lower part of the sacroiliac joint, including the mid-section of the ureter.

**Surgical technique**: For the removal of ureteral stones, we employed ureteroscopy with laser lithotripsy, which could be performed using either a semi-rigid (“Wolf” 6.5/8.5f) or a flexible (“Stortz” flexible X2) ureteroscope. Our standard surgical approach for proximal ureteral calculi involved semi-rigid ureteroscopy. In cases where the stone migrated to the kidney during the procedure, we inserted a ureteral access sheath (UAS, Cook Medical, Bloomington, IN, USA; either 9.5/11.5f or 12/14f) and proceeded with flexible ureteroscopy. We utilized both dusting (average 0.3 J × 40 Hz) and fragmentation (average 0.6 J × 6 Hz) lithotripsy techniques with a holmium laser and did not further subdivide our cohort based on the specific lithotripsy method used.

**Statistical analysis** was carried out using SPSS version 21. Continuous variables were presented as medians and interquartile ranges unless otherwise specified, while binary variables were presented with the number of observations and proportions. Group differences were assessed using the Chi-Square Test for categorical variables and the Student *t*-test/Mann–Whitney U Test for continuous variables, as appropriate. Statistical significance was defined as *p* < 0.05.

## 3. Results

### 3.1. Patients

A total of 241 patients were included in our cohort, comprising 135 (56%) with distal ureteral stones and 106 (44%) with proximal ureteral stones. The median age in our cohort was 46 years (IQR 38–64) for distal stones and 53 years (IQR 45–66) for proximal stones (*p* = 0.007). The majority of patients (72.6%) in both groups were male, with 95 (70.4%) in the distal group and 80 (75.5%) in the proximal group (*p* = 0.378). The median time from symptom onset to ureteroscopy was 14 (6–35) days in both groups. Patients with proximal ureteral stones had a slightly higher mean serum creatinine level (1.2 mg/dL) compared to those with distal stones (1.09 mg/dL, *p* = 0.044). Kidney drainage via a nephrostomy tube before ureteroscopy was observed in six (4.4%) patients with distal stones and seven (6.6%) patients with proximal stones (*p* = 0.46) (Table 1).

### 3.2. Imaging Modality

The predominant imaging modality used prior to ureteroscopy was computed tomography (CT). In the distal ureteral stones group, 78 (57.8%) patients underwent CT scans, 39 (28.9%) had ultrasonography, and 10 (7.4%) underwent both CT and ultrasonography. In the proximal ureteral stones group, 65 (61.4%) patients had CT scans, 28 (26.4%) underwent ultrasonography, and 5 (4.7%) had both CT scans and ultrasonography before ureteroscopy (Table 1).

### 3.3. Ureteral Stones

The median maximal stone diameter was 7 mm (5–10). There was a slight non-significant difference in median stone diameter between proximal (8 mm, IQR 6–10) and distal (7 mm, IQR 5–10) ureteral stones (*p* = 0.07). Stone diameter measurements were preferably derived from CT scans; however, when unavailable, ultrasonography was used. In both groups, stones were more prevalent on the left ureter, with 84 (62.2%) in the distal group and 55 (51.9%) in the proximal group. The median number of stones was one in both groups. The rate of impacted ureteral stones was higher in the proximal ureteral stones group, with 28 (26.4%) cases compared to 17 (12.6%) in the distal group (*p* = 0.006) (Table 1).

### 3.4. Ureteroscopy

The use of a ureteral access sheath (UAS) was more common in the proximal ureteral stones group, with 22 (20.8%) cases compared to 7 (5.2%) in the distal group (*p* < 0.001) (Table 1). Placement of a ureteral stent was more frequent in the proximal ureteral stones group, with 98 (92.5%) cases compared to 107 (79.3%) in the distal group (*p* = 0.004) (Table 2). The bailout rate, indicating the inability to complete ureteroscopy and stone lithotripsy, was similar in both groups, with two cases (1.88%) in the proximal ureteral stones group and one case (0.74%) in the distal ureteral stones group (*p* = 0.584) (Table 2).

### 3.5. Complications and Follow-Up

The rate of post-surgical complications was similarly low in both groups (*p* = 0.657). In the proximal ureteral stones group, complications included one case of urinary sepsis, one case of urinary tract infection, and one hospitalization within 30 days due to stent-related pain (total complication rate: 2.83%). In the distal ureteral stones group, complications comprised one case of urinary tract infection and one case of ureteral stent migration into the ureter (total complication rate: 1.48%). The median follow-up duration was 11 weeks (IQR 8–14) for distal stones and 12 weeks (IQR 10–14) for proximal stones (*p* = 0.68). SFRs were high and comparable in both groups, with 99% for distal stones and 95% for proximal stones (*p* = 1). Ultrasonography was the most frequently used imaging modality for follow-up (56.8%). The absence of ureteral stones or hydronephrosis on ultrasonography indicated a stone-free status. Ureteral stent removal occurred at a median time of 1.5 weeks (IQR 1–2) in the distal ureteral stones group and 2 weeks (IQR 1–2) in the proximal ureteral stones group (*p* = 0.085) (Table 2).

## 4. Discussion

Ureteroscopy is the preferred surgical approach for both ureteral and renal stones due to its high success rates [7,8,9,10]. However, a contentious debate exists in the urologic literature regarding the necessity of inserting a ureteral stent before definitive endoscopic treatment for proximal ureteral stones. Advocates for pre-stenting argue that it reduces operative time, lowers complications, and enhances SFRs. This argument stems from the anatomical characteristics of the ureter—a narrow, elongated tube that can be dilated by a ureteral stent, facilitating ureteroscopy [11]. Distal ureteral stones receive less benefit from pre-stenting because the ureteral orifice can be dilated during primary ureteroscopy using a semi-rigid ureteroscope or dilators [6]. In our endourological practice, we generally favor primary ureteroscopy for proximal ureteral stones, except in cases necessitating acute kidney decompression due to urinary infection.

A study by Chu L et al. [11] found that pre-stenting reduced operative time and re-operative rates in patients with large stone burdens (>1 cm) but not in those with smaller stones (<1 cm), aligning with our findings. The CORES URS global study [12], which included patients with ureteric or renal stones treated with URS worldwide, reported a 91.2% SFR following URS for ureteric stones without pre-stenting. Moreover, there was no difference in SFR between pre-stented and non-stented patients undergoing ureteroscopy for ureteral stones. Zhang J et al. [13] reported a 71% SFR after flexible URS for renal stones without pre-stenting. A systematic review and meta-analysis by Yang Y et al. [14], which included 11,138 patients, indicated an overall higher SFR in pre-stented patients. However, most studies did not subgroup patients by stone location. When stratified by stone location and size, it becomes apparent that achieving a high SFR is attainable.

Our cohort exhibited a low and comparable bailout rate (1.88% vs. 0.74% in the proximal and distal ureteral stone groups, *p* = 0.584). Most published series do not address this variable. In a multi-center study, Fuller et al. [15] reported a 7.7% overall failure rate in accessing an unstented ureter. Proximal ureteral stones had a high failure rate of 18.28%, with proximal stone location predicting failure when compared to renal stones (Odds Ratio 3.14, *p* = 0.006). Shields et al. [16] reported a higher success rate in primary ureteroscopy for proximal ureteral stones compared to nephrolithiasis, although the stone site did not predict success. In our cohort, two out of three failures to access the ureter resulted from distal ureteral strictures.

The post-surgical complication rate was not different between the two groups. Major complications, such as ureteral avulsion, urinary tract perforation, or bleeding requiring transfusion, were not observed in our cohort, consistent with findings from other studies that reported no significant differences in complication rates between stented and unstented ureters [11,12,14,17].

In our study, a ureteral stent was placed following ureteroscopy at higher rates in the proximal ureteral stones group (92.5%) and was removed after a median duration of 2 weeks. While some may consider a 2-week stent duration lengthy and potentially impacting patients’ quality of life, we follow the perspective of Makanjuola JK et al. [7], who argue that the indwelling stent time following ureteroscopy is more predictable than stenting prior to ureteroscopy.

Our study has certain limitations stemming from its retrospective design and methodology. In our institution, the common practice involves performing primary ureteroscopy without pre-stenting for all patients with proximal ureteral stones. Hence, we assumed that a comparison between primary ureteroscopy for distal ureteral stones (where pre-stenting is usually not needed) and primary ureteroscopy for proximal ureteral stones might be reasonable. Additionally, ultrasonography was the most common modality for follow-up in our cohort, which has lower sensitivity for small ureteral stones and may have contributed to our high SFR. Among the strengths of our study are stringent inclusion criteria and a standardized surgical technique within our endourology service.

## 5. Conclusions

Primary ureteroscopy with laser lithotripsy for proximal ureteral stones is a valid and feasible treatment with similar surgical outcomes, compared to that of distal ureteral stones. The SFR is high, and the complication rate is low. Hence, we conclude that preliminary ureteral stent insertion can be avoided. A randomized controlled study is needed to confirm our findings. 

## Figures and Tables

**Table 1:** Patients’ characteristics.

	Distal Ureter	Proximal Ureter	*p*-Value
**Number of patients**	135	106	
**Age (years, median, IQR)**	46 (38–64)	53 (45–66)	**0.007**
**Gender, male, n (%)**	95 (70.4%)	80 (75.5%)	0.378
**Maximal stone diameter (mm, median, IQR)**	7 (5–10)	8 (6–10)	**0.07**
**Pre-operative serum creatinine level (ng/dL) (mean, SD)**	1.09 (0.61)	1.2 (0.52)	**0.044**
**Stone side, left, n (%)**	84 (62.2)	55 (51.9)	0.107
**Pre-operative imaging**			
CT (%)	78 (57.8)	65 (61.4)	
US (%)	39 (28.9)	28 (26.4)	
CT + US (%)	10 (7.4)	5 (4.7)	
**Pre-operative nephrostomy, n (%)**	6 (4.4)	7 (6.6)	0.46
**Impacted stones, n (%)**	17 (12.6)	28 (26.4)	**0.006**
**UAS, n (%)**	7 (5.2)	22 (20.8)	**<0.001**
**Symptom duration prior to URS (days, median, IQR)**	14 (6–45)	14 (5–30)	0.19

**Table 2:** Surgical outcomes and follow-up.

Parameter	Distal Ureter (n = 135)	Proximal Ureter (n = 106)	*p* Value
**Complications (%)**	2 (1.48%)	3 (2.83%)	0.657
**Bailout (%)**	1 (0.74%)	2 (1.88%)	0.584
**SFR**	99%	95%	1
**Post-URS ureteral stent placement (%)**	107 (79.3%)	98 (92.5%)	**0.004**
**Indwelling stent time after URS (weeks, median, IQR)**	1.5 (1–2)	2 (1–2)	0.085
**Follow-up (weeks, median, IQR)**	11 (8–14)	12 (10–14)	0.068

URS = ureteroscopy; SFR = stone-free rate; IQR = interquartile range.

## Data Availability

The data presented in this study are available on request from the corresponding author. The data are not publicly available due to privacy policies.
